# Optimizing the Process Efficiency of Reactive Extrusion in the Synthesis of Vinyltrimethoxysilane-Grafted Ethylene-Octene-Copolymer (EOC-g-VTMS) by Response Surface Methodology

**DOI:** 10.3390/polym12122798

**Published:** 2020-11-26

**Authors:** Steffen Ulitzsch, Tim Bäuerle, Thomas Chassé, Günter Lorenz, Andreas Kandelbauer

**Affiliations:** 1 Center for Process Analysis & Technology (PA&T), School of Applied Chemistry, Reutlingen University, Alteburgstrasse 150, 72762 Reutlingen, Germany; steffen.ulitzsch@reutlingen-university.de (S.U.); tim.baeuerle@reutlingen-university.de (T.B.); guenter.lorenz@reutlingen-university.de (G.L.); 2 Reutlingen Research Institute (RRI), Reutlingen University, Alteburgstrasse 150, 72762 Reutlingen, Germany; 3 Institute of Physical and Theoretical Chemistry, University of Tübingen, Auf der Morgenstelle 18, 72076 Tübingen, Germany; thomas.chasse@uni-tuebingen.de

**Keywords:** reactive extrusion, silane modification, design of experiments, ethylene-octene copolymer, grafting degree, grafting efficiency, process optimization

## Abstract

Thermoplastic polymers like ethylene-octene copolymer (EOC) may be grafted with silanes via reactive extrusion to enable subsequent crosslinking for advanced biomaterials manufacture. However, this reactive extrusion process is difficult to control and it is still challenging to reproducibly arrive at well-defined products. Moreover, high grafting degrees require a considerable excess of grafting reagent. A large proportion of the silane passes through the process without reacting and needs to be removed at great expense by subsequent purification. This results in unnecessarily high consumption of chemicals and a rather resource-inefficient process. It is thus desired to be able to define desired grafting degrees with optimum grafting efficiency by means of suitable process control. In this study, the continuous grafting of vinyltrimethoxysilane (VTMS) on ethylene-octene copolymer (EOC) via reactive extrusion was investigated. Successful grafting was verified and quantified by ^1^H-NMR spectroscopy. The effects of five process parameters and their synergistic interactions on grafting degree and grafting efficiency were determined using a face-centered experimental design (FCD). Response surface methodology (RSM) was applied to derive a causal process model and define process windows yielding arbitrary grafting degrees between <2 and >5% at a minimum waste of grafting agent. It was found that the reactive extrusion process was strongly influenced by several second-order interaction effects making this process difficult to control. Grafting efficiencies between 75 and 80% can be realized as long as grafting degrees <2% are admitted.

## 1. Introduction

Ethylene-octene copolymer (EOC) is a promising material for biomaterial applications because of its favorable mechanical and interfacial properties [1,2]. Like other polyolefins, it allows adhesion of living cells on its surface and improves surface adsorption phenomena in general when present in a composite [3,4]. However, as a typical thermoplastic material, it is soluble and swellable in solvents and only of limited durability under dynamic load which currently restricts its application range.

Cross-linking improves its long-term mechanical stability and enhances further its mechanical performance. One way to achieve cross-linking of EOC is by using peroxide as an initiator for radical formation [5,6]. However, even with higher loads of peroxide, the possible crosslinking degrees are often not satisfactory [7]. Another possibility to cross-link EOC is via condensation after previous modification with a suitable silane [8]. Silane grafting with vinyltrimethoxysilane (VTMS) is the most commonly applied method in this context [9,10,11,12]. By this approach, it is possible to crosslink the VTMS modified polyolefin via a condensation reaction with silicones and, in turn, thereby increase the adhesive strength and compatibility of the silicone. The application range of both, the EOC as well as of the silicone is thereby significantly enhanced.

A convenient way to achieve VTMS grafting onto EOC on a continuous basis is reactive extrusion [13,14]. Although there have been many scientific studies on silane modification by silane grafting of EOC [15,16] or other related thermoplastics [17,18,19,20,21], this process remains difficult to control and it is still challenging to reproducibly arrive at well-defined products. It is very important to understand the effects of the individual process parameters and their synergistic action on the properties of the produced materials in order to be able to define adequate process windows that allow continuous processing of constant and defined product quality. To the best of our knowledge, as of yet, no study has dealt with the quantitative evaluation of the effects of the relevant process parameters and their synergistic interactions in a systematic way. So far, no causal mathematical model is currently available that allows predicting accurately both grafting degree and grafting efficiency in the reactive extrusion of VTMS-grafted EOC (EOC-g-VTMS).

The present study aims at filling this gap. The aim of our work is to identify and quantify the effects of the relevant process parameters governing grafting degree and efficiency. To this end, a complete face-centered experimental design (FCD) for five factors was employed. The following factors were simultaneously varied: (A) concentration of grafting monomer, (B) concentration of initiator, (C) amount of backbone polymer, (D) temperature profile in the extruder, and (E) rotational speed of the extruder screw. Successful grafting in all produced materials was demonstrated by ^1^H-NMR spectroscopy and the two responses “grafting degree” and “grafting efficiency” were determined from the integrals of the relevant signals obtained at the characteristic chemical shifts. Based on the response surface model, suitable process windows for achieving high grafting degrees and high grafting efficiencies in the reactive extrusion of VTMS and EOC are discussed.

## 2. Materials and Methods

### 2.1. Chemicals

Ethylene-octene copolymer (EOC; Engage 8200) was purchased from RESINEX Germany GmbH (Zwingenberg, Germany). Vinyltrimethoxysilane (VTMS) was kindly donated by CHT Germany GmbH (Tübingen, Germany). The peroxide 2,5-dimethyl-2,5-di(tert-butylperoxy) hexane (DTBPH; Luperox^®^ 101) was supplied from Arkema (Colombes, France). All grafting chemicals were used as received without further purification. Deuterated chloroform (99.8%) was purchased from Deutero GmbH (Kastellaun, Germany).

### 2.2. Silane Grafting by Reactive Extrusion

**Figure 1:**
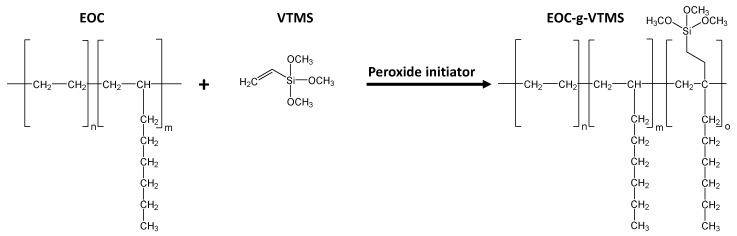
Peroxide initiated grafting reaction of vinyltrimethoxysilane (VTMS) onto ethylene-octene copolymer (EOC).

Reactive extrusion was performed in a co-rotating twin-screw extruder (Coperion ZSK 18, Stuttgart, Germany) with a screw diameter of 18 mm and a length/diameter (L/D) ratio of 48. Figure 1 shows the reaction scheme of the peroxide initiated grafting reaction of VTMS onto EOC. DTBPH was used as heat-decomposable free-radical initiator.

**Figure 2:**
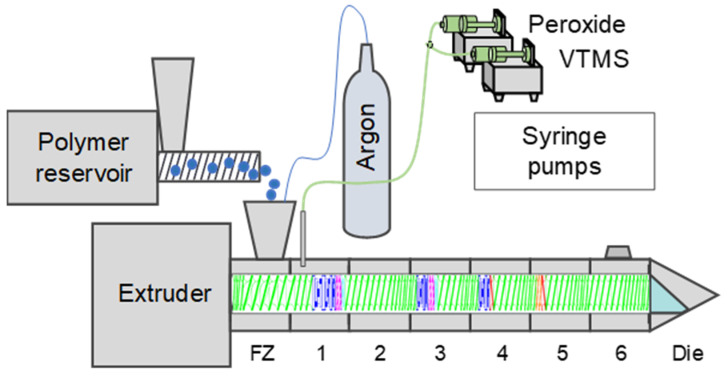
Schematic reactive extrusion setup with feeding arrangement.

Figure 2 shows schematically the reactive extrusion setup with feeding arrangement. EOC was fed using a volumetric feeder (Brabender Technologie GmbH and Co. KG, Duisburg, Germany). VTMS and DTBPH were fed into the extruder via syringe pumps at position 1. Argon was used as a protective gas to prevent side reactions of VTMS with moist air. All six temperature zones and the feeding zone (FZ) were heated and controlled separately.

During extrusion, samples were taken at 10, 15 and 20 min of continuous processing after adjusting the factor level settings to the factor level combinations as required by the FCD. Directly after sampling, the samples were placed in a vacuum drying oven at 80 °C and dried under a nitrogen stream for 24 h. This procedure ensured that excess initiator and VTMS residues were quantitatively removed. The nitrogen atmosphere was required to prevent condensation via cross-linking of the methoxy groups of the extrudate. [15]

### 2.3. Design of Experiments

A statistical experimental design (Design of Experiments, DoE) was applied to evaluate the process parameters for reactive extrusion in terms of grafting degree and grafting efficiency. A face-centered experimental design (FCD) was performed for five experimental factors. The factors studied were: VTMS feed (factor A), DTBPH feed (factor B), EOC feed (factor C), temperature increase (factor D) and extruder screw rotation speed (factor E). Their settings are listed in Table 1. The upper and lower limits (factor level range) for the settings of the VTMS and DTBPH feed rates and for the temperature (factors A, B and D) were determined by preliminary experiments. The value for the upper level of the screw rational speed (factor E) was technically limited by the torque resulting in the machinery. It was always kept below 80% to ensure homogeneous processing without mechanically damaging the processed materials. For the EOC feed rate (factor C), the lowest value which is still feasible with the machinery was chosen as the low level.

The detailed settings for the various heating segments used with the low, medium and high levels of the factor temperature increase are summarized in Table 2.

The computer program Design Expert (Version: 11.1.2.0, Stat-Ease, Inc., Minneapolis, MN, USA) was used to plan and evaluate the FCD design. Analysis of variance (ANOVA) was performed to determine the relevant factors and factor interactions. All effects having a *p*-value less than 0.05 were considered statistically significant. Only statistically significant effects were used to build the response surface model.

### 2.4. Nuclear Magnetic Resonance Spectroscopy

All samples were characterized by proton nuclear magnetic resonance spectroscopy (^1^H-NMR) measurements. Approximately 20 mg of extrudate sample were weighed into a sampling tube and dissolved in 1.0 mL deuterated chloroform (CDCl_3_). The measurements were performed on a Bruker AvanceTM III spectrometer (Bruker BioSpin GmbH, Rheinstetten, Karlsruhe, Germany) with a resonance frequency of 400.13 MHz, an acquisition time of 4.089 s, a pulse width of 14 µs and a temperature of 298 K.

For processing and analysis of the NMR spectra, the software MestreNova software (Version: 14.0.1-23559, Mestrelab Research, Santiago de Compostela, Spain) was used. All spectra were referenced to CDCl_3_ at 7.26 ppm., and phase correction, as well as baseline correction, were performed.

### 2.5. Calculation of Grafting Degree and Grafting Efficiency

**Figure 3:**
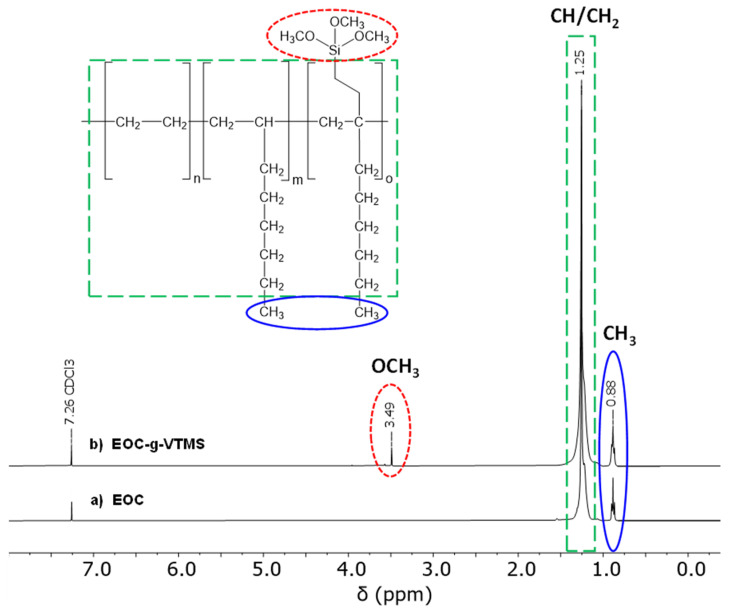
^1^H-NMR spectra of (**a**) raw unmodified EOC and (**b**) VTMS-grafted EOC (EOC-g-VTMS).

The grafting degree was calculated in weight percent (wt%) of the grafted functional group relative to the polymer weight. It was determined from the peak area integrals obtained from the ^1^H-NMR measurements using Equation (1) [22,23]. The relevant peak area integrals were: I(OCH_3_), representing the methoxy protons occurring at a chemical shift of 3.49 ppm, I(CH/CH_2_), representing the methylene protons at a chemical shift of 1.25 ppm and I(CH_3_) which represent the methyl protons at a chemical shift of 0.88 ppm (see also Figure 3). The molar masses were M(VTMS) = 148 g/mol, M(CH_2_CH_2_) = 28 g/mol, M(CH_2_CH) = 27 g/mol and M(CH_3_C) M = 27 g/mol.

(1) $$\mathrm{Grafting}\;\mathrm{degree}\;\mathrm{VTMS}\;\left(\mathrm{wt}\%\right)=\;\frac{\frac{I\left({\mathrm{OCH}}_{3}\right)}{9}\cdot M\left(\mathrm{VTMS}\right)\;\cdot 100\;\%}{\frac{I\left(\mathrm{CH}/{\mathrm{CH}}_{2}\right)}{4}\cdot M\left({\mathrm{CH}}_{2}{\mathrm{CH}}_{2}\right)-\;\frac{I\left({\mathrm{OCH}}_{3}\right)}{9}\cdot M\left({\mathrm{CH}}_{2}\mathrm{CH}\right)\;+\;\frac{I\left({\mathrm{CH}}_{3}\right)}{3}\;\cdot M\left({\mathrm{CH}}_{3}C\right)}\;\;$$

The grafting efficiency was defined as the proportion of grafting agent actually grafted onto the polymer compared to the amount of VTMS applied in the process. It is given in percent (%) as calculated from Equation (2). For this, the measured grafting degree of the VTMS grafting was divided by the grafting degree of the VTMS grafting theoretically expected if 100% of the applied reagent had reacted.

(2) $$\mathrm{grafting}\;\mathrm{efficiency}\;\left(\%\right)=\;\frac{\mathrm{measured}\;\mathrm{grafting}\;\mathrm{degree}\;\mathrm{VTMS}}{\mathrm{theoretical}\;\mathrm{grafting}\;\mathrm{degree}\;\mathrm{VTMS}}\;\cdot 100\;\%$$

## 3. Results and Discussion

### 3.1. Structural Characterization of the Reaction Products

The outcome of the peroxide-initiated grafting of VTMS onto EOC was qualitatively and quantitatively evaluated using proton nuclear magnetic resonance spectroscopy. The successful preparation of EOC-g-VTMS is supported by ^1^H-NMR data. Representative ^1^H-NMR spectra are depicted in Figure 3. Figure 3a shows the ^1^H-NMR spectrum of the unmodified EOC reference material. As expected, only signals appearing at a chemical shift of 1.25 ppm (from CH/CH_2_ protons) and 0.88 ppm (from CH_3_ protons) are present in the starting material. Figure 3b shows the ^1^H-NMR spectrum of the chemically modified EOC. After purification, the reaction product shows the same signals as the unmodified starting material. However, an additional signal at a chemical shift of 3.49 ppm (from OCH_3_, methoxy protons) is also present in the reaction product. This new signal is characteristic of the methoxy protons introduced by VTMS grafting and indicates a successful covalent modification of EOC.

### 3.2. Quantitative Determination of the Grafting Degree

The grafting degree was calculated using Equation (1) and the peak area integrals of the signals A, B, and C (see Figure 3b) for all grafting experiments. The outcome of the experimental design is summarized in Table 3. The single experimental runs comprising the overall DoE were performed in three subsequent blocks. The first block was a fractional factorial design with 3 center point (CP) experiments (Table 3, run 1–19) at intermediate factor level settings. The second set of experimental runs comprised a half fractional design intended to complete the first block to a complete full factorial design for 5 factors (Table 3, run 20–38). This second set was supplemented by three additional CPs to ensure that the two experimental blocks were indeed complementary and no systematic block effect had occurred between the two experimental series. The third series of experiments (Table 3, run 39–53) complemented the factorial design with additional so-called “axial” experiments resulting in an overall face-centered arrangement (FCD). Five further CPs again ensured compatibility of the three subsequent experimental series and showed that no time-dependent systematic effect was present and had remained unaccounted for. In total, the overall FCD covered five factors at three levels and comprised 53 experimental runs including 11 CP trials (full replicates) which ensured overall reproducibility and were used to determine the pure experimental error with high accuracy.

Samples for NMR analysis were taken during a grafting extrusion experiment always after the process had become stable upon adjusting the process parameters according to the factor levels prescribed by the experimental plan. Careful sampling with a high sampling frequency during the initial experiments had shown that the extrusion process had become stationary for practically all experiments at a sampling time of 20 min after having changed the levels of the process parameters.

The overall range of grafting degrees achieved for all experiments was between 0.23 wt% and 5.41 wt%. The grafting efficiency was between 11.95% and 85.82% with some values exceeding 100%. Values larger than 100% resulted from carry-overs from the preceding experimental runs. These experiments were not included in the model building.

### 3.3. Effect of Process Factors on Grafting Degree

The experimental design was evaluated using analysis of variance (ANOVA). All effects with a *p*-value larger than 0.05 were considered not statistically significant and, consequently, were not used for building the model. The ANOVA is shown in Table 4. To describe the data set properly, a second-order polynomial response surface model was required which was based on ten highly significant effect terms. The resulting model describes the data set very well (R^2^ = 0.989). The high value of R^2^_adjusted_ = 0.986 indicates no overfitting of the data although the model equation contained as many as ten statistically significant effect terms. R^2^_adjusted_ corrects the coefficient of determination, R^2^, by a factor that relates the number of model terms to the number of data points available from the experiment and is calculated by the formula:

$$R^{2}{}_{\mathrm{adjusted}}=1-\;\frac{\left(n-1\right)}{\left(n-p\right)}\left(1-R^{2}\right)$$

where *n* is the number of experimental runs, *p* is the number of model terms, and R^2^ is the coefficient of determination. Since the value of R^2^_adjusted_ does not deviate by much from the coefficient of determination, the used model is adequate for the analyzed data set.

The robustness of the model was further evaluated by calculating R^2^_predicted_. R^2^_predicted_ is obtained from model cross-validation. It is derived from the Predicted Residual Sum of Squares (PRESS) by finding all structurally identical polynomial models that are obtained by omitting one data point at a time and summing up all squared residuals accordingly. R^2^_predicted_ is calculated with the formula:

$$R^{2}{}_{\mathrm{prediction}}=1-\left(\frac{\sum_{i=1}^{N}{\left(y_{i}-{\hat{y}}_{i}*\right)}^{2}}{\sum_{i=1}^{N}{\left(y_{i}-\bar{y}\right)}^{2}}\right)=1-\left(\frac{\mathrm{PRESS}}{SS_{tot}}\right)$$

where $SS_{tot}$ is the sum of the squared differences between each individually measured response value, $y_{i}$, and the overall mean response value, $\bar{y}$, and PRESS is the sum of the squared differences between each individually measured response value $y_{i}$ and the predicted response value, ${\hat{y}}_{i}*$ from a model calculated by omitting the i*th experimental value for a total of N measurements. Our value for R^2^_predicted_ of R^2^_predicted_ = 0.977 is also very close to 1.00 and shows only a minor deviation from the coefficient of determination, R^2^, which indicates that the model is very robust against variations in single measurements and has high predictive power.

During data analysis, it turned out that five experimental runs had to be removed from the data set since they were identified as outliers with some distorting effect on the model. These experiments are indicated with an asterisk in Table 3. The experimental runs 4 and 34 were carried out after experiments with a VTMS feed at the highest level. In these two cases, the high VTMS levels had led to unexpected carry-overs of VTMS from the preceding experiments 3 and 33, respectively, even after 20 min of continuous processing using the adjusted settings. This had led to unintentionally high grafting efficiencies of >100%. The experimental runs 8, 11, and 15 also displayed inconsistencies. It turned out that these runs had been carried out after changes of the syringes in the pumping system. In these cases, it was observed that the pumping system had still contained some air bubbles which indicated that the process had not yet stabilized at the time the sample was taken. Therefore, all of these runs were deleted from the data set and were not considered for model building. Generally, experimental runs with comparatively low grafting degrees had displayed more difficulties with the handling.

The model contains significant non-linear terms for the factors VTMS feed (A^2^) and DTBPH feed (B^2^), which means that the grafting degree is not just simply directly proportional to the reactant concentrations but seems to be going through a maximum. The model is highly significant with an F-value of 309.90 which yields a *p*-value < 0.0001 and means that the probability for the observed deviations from linearity due just to random noise is less than 0.01%.

Table 4 makes it very clear that the grafting of VTMS onto EOC is a rather complex process. This is indicated by a large number of highly significant model terms: Four of the five factors studied showed important effects, and only the screw rotational speed seemed to be of no significant effect on the grafting degree. Not only the main factors were relevant, but also four second-order interaction (2FIA) terms turned out to be highly significant which is especially notable. Every process factor was involved in at least one synergistic interaction effect with the VTMS feed rate and the VTMS feed rate was involved in three two-factor interactions. This means that none of the effects of the studied process factors can reasonably be discussed without simultaneously considering the factor level settings of all other process factors except for the screw rotational speed. This is an important conclusion since typically, processes are still studied in a way that does not allow the detection of interaction effects at all. Most of the studies available in the literature proceed by changing the settings of a single factor at different levels while trying to keep all other factors as constant as possible in order to identify the effect of the experimentally varied factor on the targeted responses. Thereby it is tried to isolate an effect experimentally. In contrast, the approach via DoE/RSM pursued in the present contribution is based on isolating the effects of various factors simultaneously simply on a mathematical basis. This approach allows calculation of interaction effects which is not otherwise possible [24]. In the present case, it was found that the response (grafting degree) not only depended in a non-linear manner on two of the studied factors but also that the system is determined by strong interactions, making it very difficult to control in practice. In this case, appropriate process control cannot be achieved if only single factor variations are taken into account during process development and optimization.

The model equation in terms of coded factors allows identifying the relative impact of all factors by comparing the factor coefficients. The VTMS feed rate (factor A) was found to have the single strongest influence on the response “grafting degree”. It had also a strong non-linear component indicating that the net effect of this factor decreases at higher factor level settings. Factor B, the DTBPH feed, was also found to have a strong positive effect on the grafting degree and a significant negative non-linear contribution, which accounts for the gradual levelling off of the grafting degree at higher levels of DTBPH feed. Both EOC feed rate (factor C) and temperature increase (factor D) had slightly smaller influences on the grafting degree. EOC feed rate (factor C) is the only single factor effect displaying a negative linear contribution, meaning that generally, higher EOC feed rates yield lower overall grafting degrees.

All factor effects depended strongly on the factor level settings of the VTMS feed rate (factor A) since they were all involved in highly significant second-order interaction effects (Table 4, 2FIA effects of AB, AC, AD). Moreover, DTBPH and EOC feed rate also showed synergistic interaction (Table 4, 2FIA effect of BC).

The highly significant negative interaction effect of EOC feed rate with VTMS feed rate (i.e., the 2FIA effect AC) means that increasing the polymer feed when reducing the VTMS feed rate always yields lower grafting degrees than reducing the polymer feed and increasing the VTMS feed rate. This is easily understood since, of course, the grafting degree depends on the relative proportions of polymer and grafting reagent. These proportions change unfavorably when an excess of EOC relative to VTMS is employed. Hence, increasing the polymer mass throughput by increasing the EOC feed rate without appropriately adjusting the VTMS feed rate will always result in unsatisfactory grafting degrees.

More interesting is the synergistic interaction between the DTBPH and VTMS feed rates. While both the increase of the DTBPH feed rate and the increase of the VTMS feed rate lead to an increase of the grafting degree, the exact extent of the increase in grafting degree mutually depends on the settings of both parameters. For example, while an increase of the DTBPH feed rate at a high VTMS feed rate leads to a comparatively strong increase in grafting degree, the same increase of the DTBPH feed rate at a low VTMS feed rate only leads to a comparatively small increase in grafting degree. This means that at higher total concentrations of both peroxide radical initiator and crosslinking reagent, disproportionately higher grafting degrees are obtained. In addition, the interactions between VTMS and EOC feed rates (AC) and DTBPH and EOC feed rates (BC) mean that at high feed rates of both free radical initiator and silane, low polymer feed rates lead to favorable concentration ratios in the extruder, which has a positive effect on achieving high grafting degrees. High grafting degrees are thus obtained at a low polymer throughput and high use of modification reagents. In addition, a high-temperature increase rate is especially advantageous for achieving high grafting degrees at high VTMS feed rates (interaction AD) and low EOC feed rates. Under these conditions, the grafting process can be carried out with relatively long contact times of the reagents in the extruder, under favorable concentration ratios and with a high energy input into the system.

The relative magnitudes of the effect terms are summarized in the factor effects equation in terms of coded factors for the grafting degree: $\mathrm{grafting}\;\mathrm{degree}\;\mathrm{VTMS}\;\left(\mathrm{wt}\%\right)=2.8340+\;1.1701A+0.6630B-0.3900C+0.1041D+0.4483\mathrm{AB}-0.1718\mathrm{AC}+\;0.1034\mathrm{AD}-0.1112\mathrm{BC}-0.3610A^{2}-0.3510B^{2}$ For predicting quantitative values for the grafting degrees, the factor effects equation in terms of actual factors is used: $\mathrm{grafting}\;\mathrm{degree}\;\mathrm{VTMS}\;\left(\mathrm{wt}\%\right)=-0.6076+0.0555A+1.5255B+0.1152C-0.0051D+0.0112\mathrm{AB}-0.0143\mathrm{AC}+0.0003\mathrm{AD}-0.3708\mathrm{BC}-0.0002A^{2}-0.3510B^{2}$ This equation allows calculating the grafting degree within the studied range of parameter settings within a 95% confidence interval.

**Figure 4:**
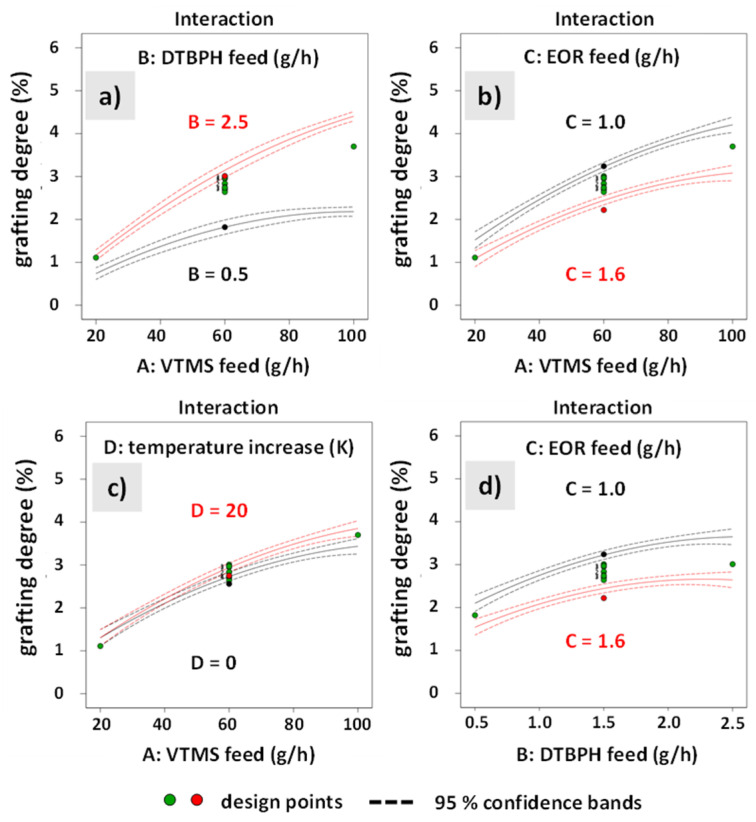
Interaction plots for each relevant interaction on the response grafting degree. The interaction effects shown are the 2 FIAs between: (**a**) VTMS and DTBPH feed rates (AB), (**b**) VTMS and EOC feed rates (AC), (**c**) VTMS feed rate and temperature increase (AD) and (**d**) DTBPH and EOC feed rates.

Figure 4 shows the interaction plots for all significant two-factor interaction effects on the response grafting degree. The solid lines show the values predicted by the model and the corresponding confidence intervals. The dots in the graph indicate the actual values from the experiments. Figure 4a shows the interaction between VTMS and DTBPH feed rates (AB). Figure 4b shows the interaction between VTMS and EOC feed rates (AC). Figure 4c shows the interaction between the VTMS feed rate and temperature increase (AD). Figure 4d shows the interaction between DTBPH and EOC feed rates.

**Figure 5:**
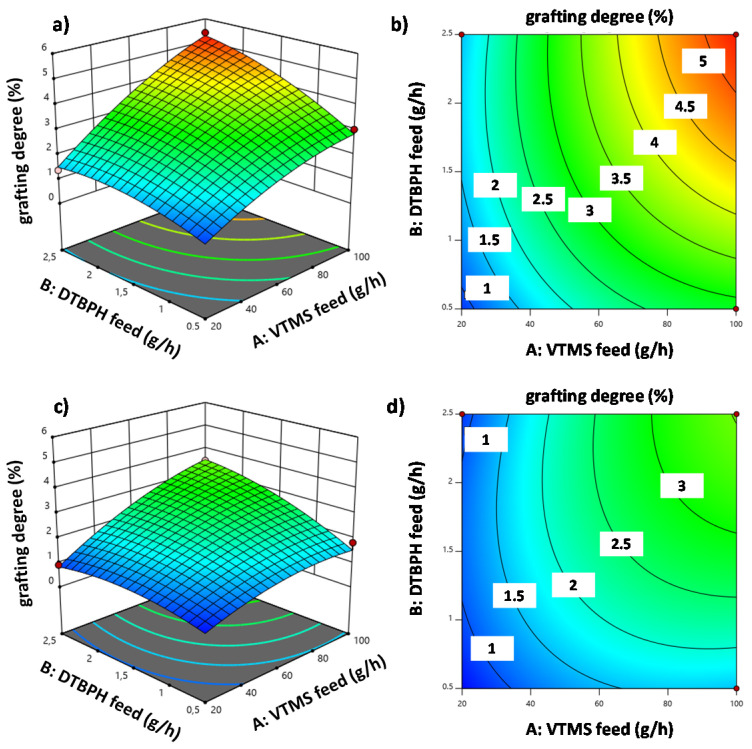
Grafting degree as a function of the VTMS and 2,5-dimethyl-2,5-di(tert-butylperoxy) hexane (DTBPH) feed rates for two scenarios: (1) 3D response surface plot (**a**) and corresponding contour line plot (**b**) at a low EOC feed and a high-temperature increase, and (2) 3D response surface plot (**c**) and corresponding contour line plot (**d**) at a high EOC feed and a low-temperature increase.

VTMS feed rate and DTBPH feed rate are the most influential factors determining the grafting degree. Figure 5 summarizes the effects of these two factors and illustrates the wide range of grafting degrees possible with reactive extrusion in 3D plots of the response surfaces and the corresponding contour line plots for two cases: (1) at a low EOC feed rate with a high-temperature increase (Figure 5a,b), and (2) at a high EOC feed rate with a low-temperature increase (Figure 5c,d).

The highest possible grafting degree achievable with this process within the factor range studied was calculated to be 5.4%. Values for the grafting degrees found in the literature for similar processes are usually in the range between 1 and 3% [21]. From the data presented above, it is evident that significantly higher grafting degrees can be obtained for EOC-g-VTMS preparation by a selection of suitable process conditions.

### 3.4. Effect of Process Factors on Grafting Efficiency

For evaluating the effect of the process parameters on grafting efficiency, as a second response, the consumption of VTMS in comparison to the quantity fed into the process was determined. Again, ANOVA was performed (Table 5). The resulting model comprised six statistically significant terms and described the data set very well as indicated by the values close to one of all three coefficients of determination (R^2^ = 0.952, R^2^_adjusted_ = 0.945, and R^2^_predicted_ = 0.919).

The VTMS and the DTBPH feed rates were the two most important factors with highly significant effect terms. DTBPH feed rate had also a highly significant non-linear effect term. EOC feed rate was less important with the grafting efficiency. The temperature increase was only weakly statistically significant as was the interaction term between temperature increase and the VTMS feed rate (2FIA effect AD). Again, screw rotational speed had no significant effect at all under the range of conditions used for the reactive extrusion experiments.

To identify the relative impact of the process factor level settings on the grafting efficiency, the model equation was calculated in terms of coded factors: $\mathrm{grafting}\;\mathrm{efficiency}\;\left[\%\right]=63.7642-9.5558A+14.4222B+1.8569C+1.4478D+\;1.5033\mathrm{AD}-9.9443B^{2}$ DTBPH feed rate (factor B) was the single most important factor determining grafting efficiency as indicated by the large values of its linear and non-linear factor effects coefficients. While the positive sign of the linear coefficient means that an increase of the DTBPH feed rate is beneficial for obtaining higher grafting efficiencies, the negative sign of the coefficient for the non-linear factor effect term indicates that the positive influence on the grafting efficiency gradually diminishes with increasing DTBPH feed rate. An increase in DTBPH feed rate leads to an increase in the concentration of radicals in the system. Therefore, it is understandable that more linkage sites are created in the polymer chain to which the silane can be effectively grafted. The non-linear term suggests that an excessive increase of the peroxide radical initiator level leads to a saturation effect due to the higher probability of side reactions such as cross-linking of the EOC or chain scission [21,25].

The VTMS feed rate is the second most important factor. It is strongly indirectly proportional to the grafting efficiency. Increasing the VTMS feed rate leads to a substantial decline in grafting efficiency. This is an important finding. While the grafting degree improves with increasing VTMS feed rate, the utilization of the raw material becomes increasingly worse. The higher the VTMS feed rate, the lower the amount of silane that is effectively incorporated into the polymer chain. This characteristic indicates that with increasing VTMS content in the extruder, an increasing proportion of the VTMS is consumed by the known side reaction of auto-polymerization of VTMS [21,26] and, hence, is no longer available for grafting.

**Figure 6:**
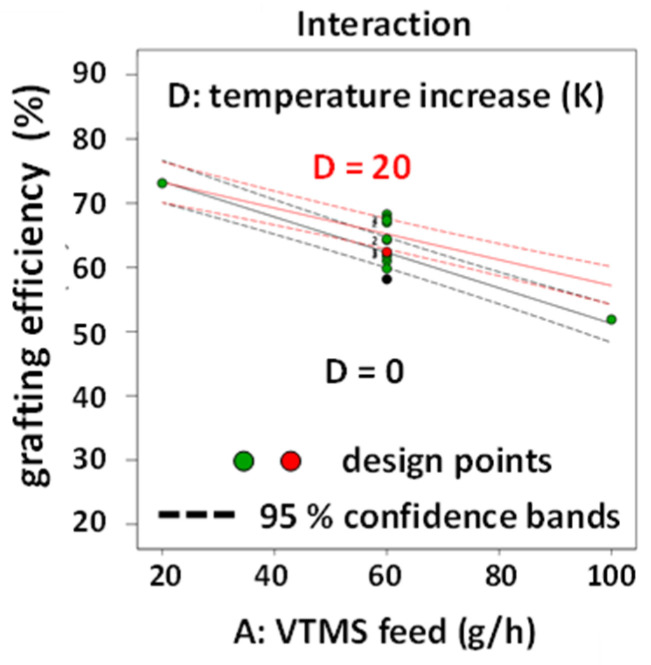
Interaction plots for each relevant interaction on the response grafting efficiency.

The effect of temperature increase (factor E) is very low. Figure 6 shows the interaction effect between VTSM feed rate and temperature increase for the response grafting efficiency. With a value of +1.5033 it is only slightly higher than the effect of temperature increase with +1.4478 and much smaller (roughly, by a factor of 10) than the most relevant factor effect DTBPH feed rate (+14.4222). Hence, its influence on distorting the resulting 3D response surface is rather small.

**Figure 7:**
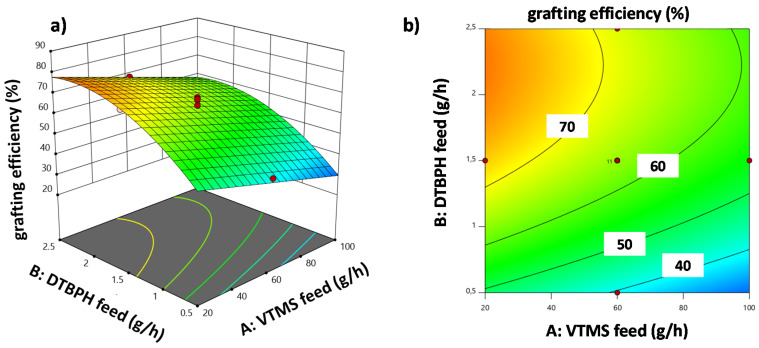
(**a**) 3D response surface plot and (**b**) contour plot for the response grafting efficiency with the correlation of VTMS feed and DTBPH feed and all other factors at intermediate factor level settings.

The grafting efficiency can be calculated using the following equation in actual factor level units: $\mathrm{grafting}\;\mathrm{efficiency}\;\left[\%\right]=26.8506-0.2765A+44.2550B+6.1896C-0.0807D+\;0.0038\mathrm{AD}-9.9443B^{2}.$ The combined action of VTMS and DTBPH feed rates on the grafting efficiency is illustrated for intermediate factor level settings of all other factors in the 3D response surface diagram shown in Figure 7a and the corresponding contour line plot (Figure 7b).

Detailed data on the grafting efficiency in EOC-g-VTMS grafting are not available from the literature to the best of our knowledge. However, similar grafting reactions are described to yield utilization rates of up to ca. 60% [21]. The highest possible grafting efficiency of the EOC-g-VTMS grafting process achieved here was >80%. Figure 7 shows that there is a broad region of possible factor level combinations available in the design space that results in grafting efficiencies well beyond 60%.

### 3.5. Process Windows for the Grafting Reaction via Reactive Extrusion

The two target responses “grafting degree” and “grafting efficiency” are influenced differently and partly in opposite directions by the investigated process factors. While a high VTMS feed rate improved the achievable grafting degrees, the grafting efficiency, in contrast, declined. It is not possible to achieve both target variables completely satisfactorily at the same time, i.e., the process cannot achieve both a high grafting degree and a high grafting efficiency simultaneously. To operate the process satisfactorily, a compromise must be found between the two target responses in each case. Grafting degree and grafting efficiency depend in a rather complex way on the set levels of all process parameters and the settings of all factors need to be defined in a highly coordinated way in order to achieve desirable pre-defined grafting degrees and grafting efficiencies in the reactive extrusion process. From the analysis of the factors so far, the quantitative correlations for both responses are known. In the next step, the two models are used to optimize both target responses and define suitable process windows.

Both mathematical models for predicting the grafting degree and the grafting efficiency are very appropriate as reflected by the values close to 1.00 of the various coefficients of determination R^2^, R^2^_adjusted_ and R^2^_predicted_. From the models, it becomes clear that high DTBPH and EOC feed rates are favorable in order to achieve both high grafting degrees and high grafting efficiencies. When low levels in DTBPH feed rates are employed, generally inferior grafting efficiencies and low grafting degrees are obtained that cannot be compensated for by adjustments in the other parameter settings. Since DTBPH acts as a radical starter initiating the grafting reaction, this is understandable.

**Figure 8:**
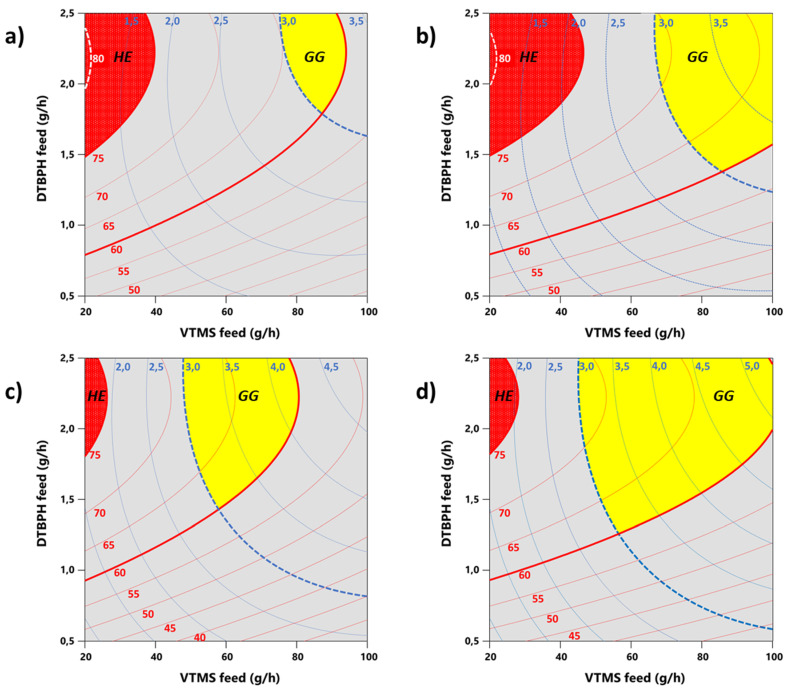
Overlay contour plots illustrating process windows in the design space for various scenarios in the grafting of VTMS onto EOC via reactive extrusion. Process windows for high grafting efficiency (HE regions, indicated in red) and good grafting (GG regions, indicated in yellow) are defined in dependence of grafting degree and grafting efficiency on the VTMS and DTBPH feed rates at (**a**) low-temperature increase and high EC feed rate, (**b**) high-temperature increase and high EOC feed rate, (**c**) low-temperature increase and low EOC feed rate, and (**d**) high-temperature increase and low EOC feed rate. Red dotted lines indicate settings of process parameters that yield the same grafting efficiency. The minimum required grafting efficiency is indicated by the bold red line. Blue dotted thin lines indicate settings of process parameters that yield the same grafting degrees. A minimum grafting degree of 3% is indicated by the bold dotted blue line.

At a high level of DTBPH feed rate, the VTMS feed can conveniently be used to adjust the process to yield the desired grafting degree and grafting efficiency. The rotational speed of the extruder screw has no influence on the outcome of the process. To be efficient, the silane reagent should always be grafted at least to an extent larger than 60%. This means that only less than 40% of the employed grafting agent remains as “leftover monomer” which needs to be removed during a subsequent purification step. Figure 8 shows overlay contour plots that allow defining process windows for various scenarios in the grafting of VTMS onto EOC via reactive extrusion.

Two main desirable regions of process factor level combinations that lead to satisfactory grafting results can be identified in the design space: a region with high grafting efficiency and a region with a good grafting degree. Figure 8a–d show the process windows for high grafting efficiencies (“High Efficiency” regions, HE, colored in red, located in the upper left corner of the design space) and for good grafting degrees (“Good Grafting” regions, GG, colored in yellow, located in the upper right corner of the design space) in dependence of the VTMS and the DTBPH feed rates. Four scenarios are shown: (1) for the high level of EOC feed rate and low-temperature increase (Figure 8a), (2) for the high level of EOC feed rate and high-temperature increase (Figure 8b), (3) for the low level of EOC feed rate and a low-temperature increase (Figure 8c), and (4) for the low level of the EOC feed rate and the high-temperature increase (Figure 8d). The HE regions include all process parameter settings that lead to utilization rates of VTMS higher than 75%. When defining these process windows, no restrictions were made with regard to the grafting degrees achievable under these process conditions. The GG regions include all process parameter settings that result in grafting degrees greater than 3% (see the blue dotted line in the overlay contour plot at the 3% level). At the same time, the minimum requirement in grafting efficiency of 60% was specified as an additional constraint. This lower limit is indicated by the bold red line in the diagram at the 60% grafting efficiency contour line.

The highest possible grafting efficiencies are achieved at a higher level of the EOC feed rate. Hence, when the process is performed at a high mass throughput in the polymer material to be grafted, process efficiency in terms of VTMS consumption is automatically relatively high up to 80% (Figure 8a,b). Working at high-temperature increase profiles further extends the range of useful process parameter settings that lead to high process efficiencies towards higher grafting degrees (see the broader process window in Figure 8b). Thereby, higher grafting degrees are possible while still maintaining relatively high grafting efficiencies (Figure 8b). In contrast, applying lower temperature increases and at the same time reducing the EOC feed rate (Figure 8c) leads to generally lower grafting efficiencies and a comparatively more restricted process window for good grafting degrees at reasonable grafting efficiency.

Figure 8d illustrates that the highest possible grafting degrees approach values close to 5% and more. However, such high grafting degrees can only be achieved when accepting a grafting efficiency only slightly above the minimum requirement of 60%. Moreover, the process needs to be performed at the lower end of the spectrum of EOC feed rates (smaller mass throughput in polymer) and at the high level of temperature increase. This makes the process generally more costly when high grafting degrees are aimed for.

If a higher percentage of raw material is to be utilized in the process and a correspondingly smaller excess of grafting reagent is permitted, only lower grafting degrees can be obtained with this manufacturing procedure. If it is aimed at grafting degrees around 3%, the process can be carried out with an efficiency well around 70% under a variety of appropriate manufacturing conditions. The required settings for the process factors are discernable from the diagrams given in Figure 8. Only low-temperature increases and high EOC feed rates need to be avoided.

Figure 8 clearly shows the possibilities and limitations of the reactive extrusion process for the grafting of VTMS onto EOC. Certainly, the higher the grafting efficiency is, the more cost-effective and environmentally compatible the whole process becomes. However, for better raw material utilization a compromise must be accepted regarding the achievable grafting degrees.

Both grafting efficiency and grafting degree depend very much on DTBPH and VTMS feed rate in a non-linear way. Additionally, there are strong second-order interactions between these two and the other factors. This causes the target responses to be influenced by the process conditions in a complicated manner. For instance, the grafting efficiency can always be significantly increased by increasing the DTBPH feed rate while maintaining the same VTMS feed rate. It always increases by about 15% when the DTBPH feed rate is doubled. Similarly, the grafting degree also always increases with increasing the DTBPH feed rate. However, the extent to which the latter is increased depends on all other process parameter settings as well simultaneously. If, for example, at a low-temperature increase and low EOC feed rate, the DTBPH feed rate is doubled from 1 g/h to 2 g/h at a VTMS feed rate of 40 g/h, the grafting efficiency of the process is increased from approx. 55–60% to significantly more than 70% (Figure 8c). This is accompanied by a simultaneous moderate increase in the grafting degree from approx. 2% to 2.6% by roughly 0.6%. In contrast, at a VTMS feed rate of 80 g/h, the grafting degree increases by significantly more than 1% from approx. 3% to >4% while the grafting efficiency at these settings again increases by ca. 15%. Unfortunately, the grafting efficiency is significantly worse at higher VTMS feed rates than at low ones. Figure 8c shows that doubling the VTMS feed rate from 40 to 80 g/h reduces the grafting efficiency at 1g/h DTBPH feed rate to about 45%. This means that at twice the VTMS feed rate, doubling the DTBPH feed rate does not result in any net gain in process efficiency but mainly compensates for the loss in grafting efficiency brought about by increasing the VTMS feed rate. In other words, at higher grafting degrees, the generally poorer grafting efficiency can only be insufficiently increased by increasing the chemical dosing rates. This automatically leads to a systematically more inefficient process if higher grafting degrees are targeted, and the decline in grafting efficiency can no longer be compensated for satisfactorily by suitable selection of the settings of the control variables.

## 4. Conclusions

In this work, the effects of the five process factors VTMS feed rate, DTBPH feed rate, EOC feed rate, temperature increase along the extruder segments and screw rotational speed on the grafting degree and the grafting efficiency of the grafting of VTMS onto EOC via reactive extrusion were quantitatively studied using a face-centered experimental design strategy. Factor effect estimates were determined for all factors and their interactions. For both target responses, linear and non-linear dependencies on the process factors were found and several highly significant two-factor interaction effects were identified to have a strong impact on the outcome of the process. Although the VTMS and DTBPH feed rates had the most pronounced effects, the second-order interactions were of the same order of magnitude. This explains why it is difficult to control the process in such a way that it delivers defined output in quality.

Statistically highly significant and accurate response surface models for both the grafting degree and the grafting efficiency were derived and applied to deduce suitable process windows for simultaneously optimizing the two target responses. Process conditions were found for achieving EOC-g-VTMS grafting degrees as high as >5 wt% VTMS (GG region in the design space) or grafting efficiencies up to >80% (HE region in the design space). However, it was shown that it is not possible to arrive at maximum values for both responses at the same time and a compromise must be made.

This study demonstrates that the relative concentrations of the employed chemicals VTMS, DTBPH and EOC present in the extruder had altogether more influence on the process outcome than the machine parameters temperature profile and screw rotational speed. Hence, they are well suited to control and define the process outcome. It is reasonable to assume that related processes performed by reactive extrusion follow similar patterns and may be controlled in an analogous way mainly by controlling the stoichiometric relationships. The procedure described and illustrated here can therefore be very helpful to quickly find suitable process windows for related processes.

## Figures and Tables

**Table 1:** Factors studied and factor levels used in the face-centered experimental design (FCD).

Factor	Name	Unit	Low Level (−1)	Center Point (0)	High Level (+1)
A	VTMS feed	g/h	20	60	100
B	DTBPH feed	g/h	0.5	1.5	2.5
C	EOC feed	kg/h	1.0	1.3	1.6
D	Temperature increase	K	0	10	20
E	Extruder screw rotational speed	rpm	100	150	200

**Table 2:** Factors studied and factor levels used in the FCD.

Setting	Temperature Increase	Unit	Segment 1	Segment 2	Segment 3	Segment 4	Segment 5	Segment 6
(−1)	0	°C	100	120	150	180	200	200
(0)	10	°C	110	130	160	190	210	210
(+1)	20	°C	120	140	170	200	220	220

**Table 3:** Experimental data for response surface analysis of the face-centered experimental design with three-level factor settings for the five factors. The experiments are shown in the randomized order the runs were actually performed. Experiments were conducted in three blocks: Block 1: Runs 1 to 19. Block 2: Runs 20 to 38. Block 3: Runs 39 to 53.

Run	Factor Level Settings					Response Variables	
	A VTMS Feed Rate (g/h)	B DTBPH Feed Rate (g/h)	C EOC Feed Rate (g/h)	D Temp. Increase (K)	E Screw Rotational Speed (rpm)	Grafting Degree (wt%)	Grafting Efficiency (%)
1	20	0.5	1.0	0	200	0.91	46.20
2	100	2.5	1.6	0	100	3.48	59.25
3	100	0.5	1.0	0	100	2.14	23.54
4	20	0.5	1.6	0	100	1.52 *	123.06 *
5	100	0.5	1.6	0	200	1.48	25.19
6	100	2.5	1.0	0	200	4.71	51.89
7	20	2.5	1.0	0	100	1.68	85.82
8	20	2.5	1.6	0	200	0.57 *	46.60 *
9	60	1.5	1.3	10	150	2.95	66.98
10	100	2.5	1.0	20	100	5.41	59.61
11	20	2.5	1.0	20	200	0.38 *	19.43 *
12	100	0.5	1.6	20	100	2.04	34.67
13	100	0.5	1.0	20	200	2.72	29.95
14	20	2.5	1.6	20	100	0.94	76.26
15	20	0.5	1.0	20	100	0.23 *	11.95 *
16	20	0.5	1.6	20	200	0.59	47.77
17	60	1.5	1.3	10	150	3.01	68.25
18	100	2.5	1.6	20	200	4.03	68.63
19	60	1.5	1.3	10	150	2.98	67.65
20	60	1.5	1.3	10	150	2.97	67.31
21	20	0.5	1.0	20	200	0.99	50.60
22	100	2.5	1.0	20	200	5.22	57.57
23	100	2.5	1.6	20	100	3.97	67.56
24	20	0.5	1.6	20	100	0.72	58.35
25	100	0.5	1.6	20	200	1.87	31.81
26	100	0.5	1.0	20	100	3.03	33.30
27	20	2.5	1.0	20	100	1.37	70.04
28	20	2.5	1.6	20	200	1.03	83.24
29	20	2.5	1.0	0	200	1.46	74.64
30	100	2.5	1.0	0	100	5.24	57.76
31	100	2.5	1.6	0	200	3.49	59.40
32	100	0.5	1.6	0	100	1.83	31.06
33	100	0.5	1.0	0	200	2.59	28.47
34	20	0.5	1.0	0	100	2.70*	137.90*
35	20	0.5	1.6	0	200	0.63	51.40
36	20	2.5	1.6	0	100	0.91	74.18
37	60	1.5	1.3	10	150	2.69	61.00
38	60	1.5	1.3	10	150	2.72	61.66
39	60	1.5	1.3	10	150	2.64	59.81
40	60	1.5	1.3	10	150	2.83	64.26
41	60	1.5	1.3	0	150	2.56	58.16
42	20	1.5	1.3	10	150	1.11	73.10
43	60	2.5	1.3	10	150	3.01	68.40
44	60	1.5	1.6	10	150	2.22	61.41
45	60	0.5	1.3	10	150	1.82	41.34
46	60	1.5	1.3	20	150	2.75	62.41
47	60	1.5	1.3	10	150	2.84	64.40
48	100	1.5	1.3	10	150	3.70	51.88
49	60	1.5	1.0	10	150	3.24	57.37
50	60	1.5	1.3	10	200	2.80	63.42
51	60	1.5	1.3	10	100	2.72	61.65
52	60	1.5	1.3	10	150	2.75	62.32
53	60	1.5	1.3	10	150	2.74	62.15

* not used for the model.

**Table 4:** ANOVA for response surface analysis of grafting degree.

Source	Degree of Freedom (df)	Sum of Squares	Mean Square	F-Value	*p*-Value
Block	0.7334	2	0.3667	-	-
Model	67.29	10	6.73	309.90	<0.0001
A-VTMS feed	37.43	1	37.43	1724.02	<0.0001
B-DTBPH feed	12.13	1	12.13	558.81	<0.0001
C- EOC feed	4.06	1	4.06	187.21	<0.0001
D-temperature increase	0.2896	1	0.2896	13.34	0.0008
AB	5.16	1	5.16	237.69	<0.0001
AC	0.7328	1	0.7328	33.75	<0.0001
AD	0.2656	1	0.2656	12.23	0.0013
BC	0.3288	1	0.3288	15.15	0.0004
A^2^	0.4421	1	0.4421	20.36	<0.0001
B^2^	0.4180	1	0.4180	19.25	0.0001
Residual	0.7599	35	0.0217	-	-
Lack of Fit	0.6847	27	0.0254	2.70	0.0732
Pure Error	0.0753	8	0.0094	-	-
Cor Total	68.78	47	-	-	-

**Table 5:** Analysis of variance (ANOVA) for response surface analysis of grafting efficiency.

Source	Degree of Freedom (df)	Sum of Squares	Mean Square	F-Value	*p*-Value
Block	343.84	2	171.92	-	-
Model	9867.15	6	1644.52	129.81	<0.0001
A-VTMS feed	2516.78	1	2516.78	198.66	<0.0001
B-DTBPH feed	5983.63	1	5983.63	472.30	<0.0001
C-EOC feed	97.02	1	97.02	7.66	0.0086
D-temperature increase	57.57	1	57.57	4.54	0.0394
AD	57.72	1	57.72	4.56	0.0391
B^2^	652.03	1	652.03	51.47	<0.0001
Residual	494.10	39	12.67	-	-
Lack of Fit	455.17	31	14.68	3.02	0.0527
Pure Error	38.93	8	4.87	-	-
Cor Total	10705.08	47	-	-	-
